# Collaborative interactions to enhance gas binding energy in porous metal–organic frameworks

**DOI:** 10.1107/S2052252517001683

**Published:** 2017-02-23

**Authors:** Rui-Biao Lin, Banglin Chen

**Affiliations:** a Department of Chemistry, University of Texas at San Antonio, San Antonio, TX 78249-0698, USA

**Keywords:** metal–organic frameworks, hydrogen storage, binding affinity, tailored pore geometry, dispersive interactions

## Abstract

Metal–organic frameworks (MOFs) are potentially useful materials for hydrogen and methane storage. However, the weak interactions between the MOF host and gas guest molecules have limited their storage capacities at elevated temperatures. In this issue, Alkordi *et al.* [*IUCrJ* (2017), **4**, 131–135] illustrate an example of a porous MOF with a suitable pore size and unique pore surface for enhanced interaction with hydrogen molecules, providing the promise of further increasing the gas binding affinity through collaborative interactions.

Clean and renewable energy and technologies are in great demand due to environmental and energy concerns. Hydrogen (H_2_) and natural gas (NG, mainly methane) are more environmentally friendly and efficient alternative fuels given their lower exhaust emissions and high gravimetric energy densities. Compared with conventional fossil fuels, the combustion of hydrogen emits only water, while natural gas emits 25% less carbon dioxide. For hydrogen and methane, their gravimetric heats of combustion (123 and 55.7 MJ kg^−1^, respectively) are higher than that of gasoline (47.2 MJ kg^−1^) (Suh *et al.*, 2012 ▸). These advantages make hydrogen and methane promising clean power sources. However, the density of either hydrogen or methane is extremely low, making their on-board storage a great challenge and preventing the widespread use of these gases in vehicles. Possible solutions in conventional storage could be liquefaction at low temperature (20 K for liquefied hydrogen) or compression at ambient temperature and high pressure (hundreds of atmospheres). These solutions require heavy bulky fuel tanks or expensive compressors, putting a serious limitation on their possible applications, particularly for passenger vehicles. An alternative solution is to increase the gas storage density under relatively mild conditions using porous materials. Introducing porous materials into fuel tanks can drastically reduce the stored pressure at ambient temperature, which is more efficient and safe. Hence it is critically important to develop efficient adsorbent materials to use these promising clean fuels.

Metal–organic frameworks (MOFs, also known as porous coordination polymers) are a new generation of porous materials (Furukawa *et al.*, 2013 ▸) which have intensively changed a wide range of technological fields, especially in the separation and storage of gases. Owing to their uniform pore structures, tunable pore sizes and designable structures, MOFs have been proven as ideal candidates for hydrogen and methane storage (Suh *et al.*, 2012 ▸; Li *et al.*, 2016 ▸; He *et al.*, 2014 ▸; Mason *et al.*, 2014 ▸; Sculley *et al.*, 2011 ▸). Benefitting from the infinite permutations of their inorganic and organic components, MOFs are more extensive in their diversity than any other class of conventional porous material. The pore structures are easily controlled to obtain optimized performance, which has been clearly demonstrated in the latest important progress on MOFs for hydrocarbon separation (Cui *et al.*, 2016 ▸). MOFs were first reported to store hydrogen gas in around 2003 (Rosi *et al.*, 2003 ▸), while methane storage can be dated back to 1997 (Kondo *et al.*, 1997 ▸). Since then, remarkable progress has been achieved and hundreds of MOFs have been developed with a high capacity for these fuel gases.

Generally, the interaction between hydrogen and the surface of a MOF is quite weak, so functionalizing the organic linkers will have little positive effect on hydrogen adsorption. By increasing the pore volume and surface area, the total gravimetric hydrogen capacity can be up to 17.6 wt% when using MOF-210 as adsorbent but at a very low temperature of 77 K (Furukawa *et al.*, 2010 ▸). There is still no MOF material which can meet the US Department of Energy targets (5.5 wt%, 40 g l^−1^ at 233–333 K and below 100 bar) for hydrogen adsorption (Suh *et al.*, 2012 ▸). It is very important to increase the hydrogen binding energy to store hydrogen at a comparatively high temperature. One of the more efficient approaches is to immobilize open metal sites for their stronger interactions with hydrogen molecules (Chen *et al.*, 2005 ▸, 2008 ▸; Zhou *et al.*, 2008 ▸). For instance, M′MOF-1 has very high initial adsorption enthalpy of 12.3 kJ mol^−1^ by using a metalloligand with an open Cu^II^ site (Chen *et al.*, 2008 ▸), while that of MOF-74(Ni) is 12.9 kJ mol^−1^ (Zhou *et al.*, 2008 ▸). These are among the best values reported to date. However, it is still a daunting challenge to increase the hydrogen binding energy further, although the ideal hydrogen binding affinity is suggested to be about 20–40 kJ mol^−1^ to meet the DOE target (Lochan & Head-Gordon, 2006 ▸).

The paper published in this issue of **IUCrJ** by Eddaoudi and co-workers (Alkordi *et al.*, 2017 ▸) demonstrates that the common weak hydrogen binding affinities to aromatic surfaces can be dramatically enhanced, and this is accomplished by forcing the gas molecules to form multiple dispersive interactions with the specific pore geometry of a new MOF (Fig. 1 ▸). This MOF is a three-dimensional open framework, in which infinite lead(II) carboxylate chains are connected by dicarboxylate linkers to form narrow one-dimensional square-like channels (pore aperture size 0.4 nm). There are only exposed aromatic rings in a specific array on the pore surface. However, sorption measurements showed that the hydrogen sorption isotherm exhibits a rare type I character with a sharp increase at low pressure. Importantly, the adsorption enthalpy for hydrogen was calculated to be about 9 kJ mol^−1^, which is more than twice that for a hydrogen molecule interacting with a benzene ring (about 3.4–4.0 kJ mol^−1^) and higher than the values of about 7 kJ mol^−1^ generally found in porous MOFs. This is attributed to multiple weak intermolecular forces between a single hydrogen molecule and the pore surface which are enforced through collaborative interactions confined in a suitable pore space of about 0.4 nm.

This work provides a unique example of a porous MOF with a suitable pore size and surface for collaborative interaction with hydrogen molecules, thus enhancing the hydrogen binding affinity. It will initiate extensive research efforts on this very important topic. If other specific sites such as open metal sites can be simultaneously incorporated into such unique pore surfaces, the additionally immobilized sites might further enhance their interaction with gas molecules, thus paving the way to target MOF materials for hydrogen storage at elevated temperatures. Of course, MOFs still need high densities of such unique pore spaces and moderately high porosities to secure their high gas storage capacities.

## Figures and Tables

**Figure 1:**
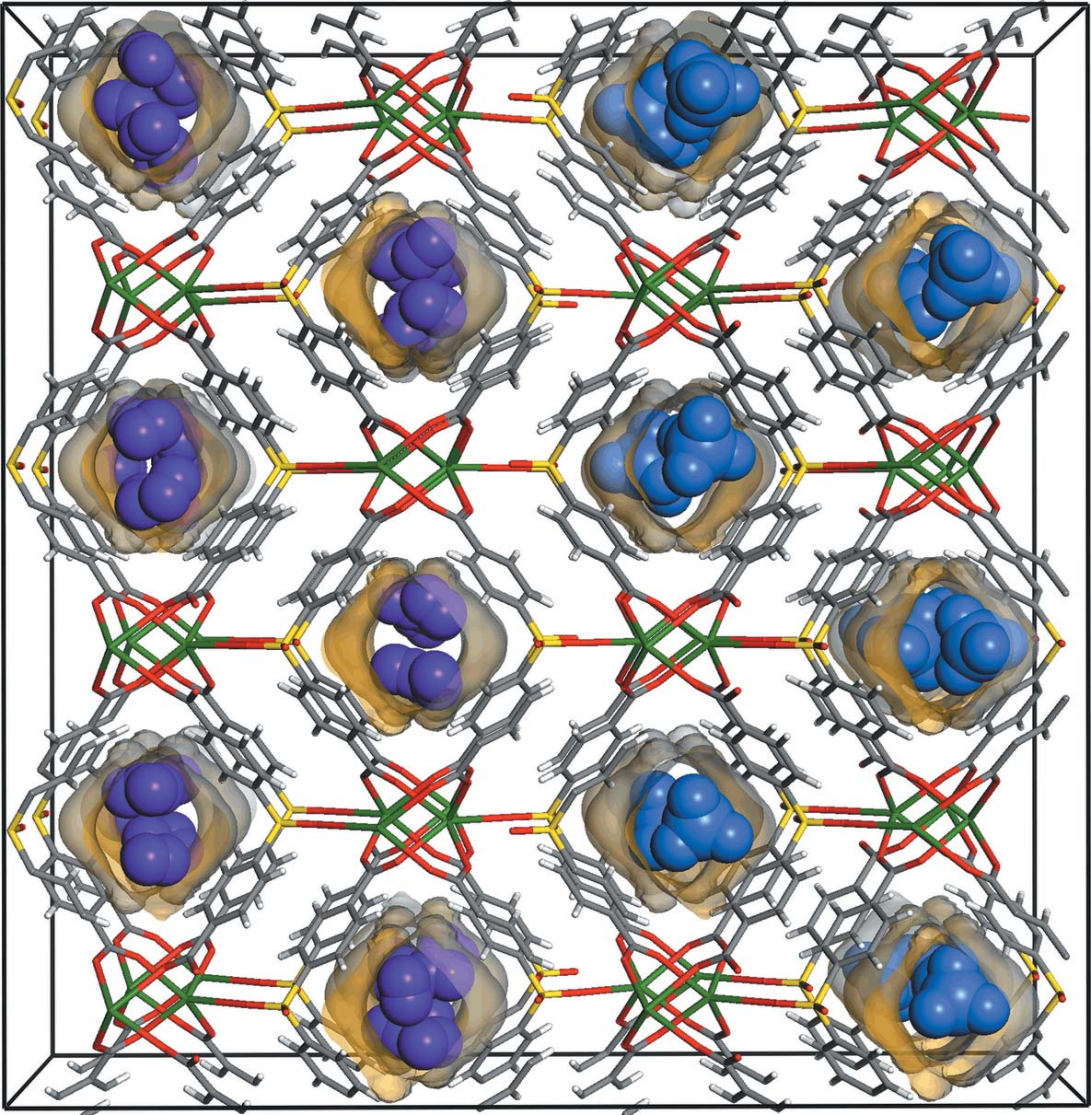
A schematic diagram of hydrogen and methane storage in the pores of the lead(II) carboxylate MOF, viewed parallel to [100] (carbon atoms are colored gray, oxygen red, sulfur yellow and lead green, while hydrogen and methane guest molecules are highlighted in violet and light blue, respectively).
